# Hotspot Selective Preference of the Chimeric Sequences Formed in Multiple Displacement Amplification

**DOI:** 10.3390/ijms18030492

**Published:** 2017-02-24

**Authors:** Jing Tu, Na Lu, Mengqin Duan, Mengting Huang, Liang Chen, Junji Li, Jing Guo, Zuhong Lu

**Affiliations:** State Key Lab of Bioelectronics, School of Biological Science and Medical Engineering, Southeast University, Nanjing 210096, China; jtu@seu.edu.cn (J.T.); nlu@seu.edu.cn (N.L.); 220163884@seu.edu.cn (M.D.); 213133851@seu.edu.cn (M.H.), tongust@163.com (L.C.); 230139150@seu.edu.cn (J.L.); lebrowngj@126.com (J.G.)

**Keywords:** chimeras, chimeric hotspots, multiple displacement amplification (MDA), φ29 polymerase

## Abstract

Multiple displacement amplification (MDA) is considered to be a conventional approach to comprehensive amplification from low input DNA. The chimeric reads generated in MDA lead to severe disruption in some studies, including those focusing on heterogeneity, structural variation, and genetic recombination. Meanwhile, the generation of by-products gives a new approach to gain insights into the reaction process of φ29 polymerase. Here, we analyzed 36.7 million chimeras and screened 196 billion chimeric hotspots in the human genome, as well as evaluating the hotspot selective preference of chimeras. No significant preference was captured in the distributions of chimeras and hotspots among chromosomes. Hotspots with overlaps for 12–13 nucleotides (nt) were most likely to be selected as templates in chimera generation. Meanwhile, a regularly selective preference was noticed in overlap GC content. The preferences in overlap length and GC content was shown to be pertinent to the sequence denaturation temperature, which pointed out the optimization direction for reducing chimeras. Distance preference between two segments of chimeras was 80–280 nt. The analysis is beneficial for reducing the chimeras in MDA, and the characterization of MDA chimeras is helpful in distinguishing MDA chimeras from chimeric sequences caused by disease.

## 1. Introduction

After the advent of multiple displacement amplification (MDA) in 2002 [1], this isothermal amplification method has been used in a series of applications, including nucleic acid amplification from a single sperm [2,3,4], an oocyte [5], and even a part of the chromosomes of a single cell [6,7], as well as haplotype analysis based on dilution and amplification [8,9]. Nowadays, MDA is considered to be a conventional approach in comprehensive amplification from low input DNA, prior to constructing libraries of next generation sequencing. In most MDA, φ29 DNA polymerase is used with random hexamer primers to amplify DNA in a cascading, strand displacement reaction [1]. Due to its high processivity and proofreading activity, φ29 polymerase is able to generate large DNA fragments (>10 kilo-base) by using small starting materials (100 fg–10 ng) [10].

Along with the development and application of MDA, chimeric reads (i.e., chimeras) were discovered and noticed in the MDA-associated sequencing data [11,12,13,14]. The chimeric artifacts are DNA rearrangements in amplified DNA, which are derived from the MDA procedure [11,12]. A chimera consists of two or more parts which locate inconsecutively but adjacently on a chromosome [13]. Although chimeric reads can be filtered, they still lead to disruption in some studies, including those focusing on heterogeneity, structural variation, and genetic recombination. In-depth characterization of chimerism is beneficial for reducing this disruption. Meanwhile, since chimeras are produced in the enzymatic reaction of φ29 polymerase, the analysis of its by-products gives a new approach to gain insights into the reaction process.

**Figure 1:**
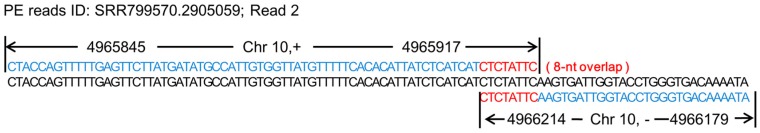
A chimeric read with the two segments located on the complementary strands and an 8-nucleotide (nt) overlap. The chimeric read is 101 base pairs (bp) in length. The two segments are mapped to the different strands of chromosome 10 and have an 8-nt overlap. In the sense strand, the sequences of the 8-nt overlap in the two locations are reverse complementary sequences; CTCTATTC and GAATAGAG. The sequence of segments are drawn in red and blue, and the red parts are the sequences of overlap.

In 2007, Lasken and Stockwell discovered 475 MDA chimeras in sequencing data of *Escherichia coli* using 454 sequencing platform [12]. Two major types of chimeras; chimeras with inverted sequences and chimeras with direct sequences, were characterized in this work. They also illustrated the mechanism of chimera formation with inverted sequences. However, hundreds of chimeras are far from sufficient to be representative. Chimerism happening in human genome sequencing data could not be equivalently reflected by the genomic simplicity of the *E. coli*. In previous work [13], we performed a chimerism analysis in over 130 giga-base human MDA sequencing data, which were used for whole genome haplotype assembling [9]. Over 40 million chimeric reads were discovered and the chimerism was investigated. The probable mechanisms of chimera formation were illustrated based on the hyperbranched structure of the MDA products. One-level chimeras with two inverted sequences were revealed to be the major chimeric type [13]. Chimeras that consist of two rearranged segments are called 1-level chimeras (Figure 1). The genomic distances of the two segments are mainly within 5 kilo-nucleotide (knt) [13]. Almost all chimeras have an overlapped sequence in the tail of the former segment and the head of the following segment (Figure 1). For the chimeras with inverted sequences, two segments locate on complementary strands, while the overlapped is sequence located on the two complementary strands simultaneously. In fact, in the same strand, two regions of an overlap are reverse complementary. Taking the chimera in Figure 1 an example, in the sense strand sequences of the 8-nt overlap in the two locations are CTCTATTC and GAATAGAG. These reverse complementary sequence pairs are different in length and mainly locate within 5 knt. Based on the probable mechanisms of chimera formation, each reverse complementary sequence pair in a strand is a potential template of chimeric reads. Here, we name these reverse complementary sequence pairs chimeric hotspots. Each chimeric hotspot is made up of a pair of reverse complementary sequences in the same DNA strand. The hotspots here are potential templates of chimeras which are generated in MDA procedure, and are different from the other types of hotspots in the genome, such as recombination hotspots [15] and functional hotspots [16]. In the human genome, there are numerous chimeric hotspots, especially hotspots with short overlaps. Chimeras ought to be generated by any of these chimeric hotspots. However, based on abundant sequencing data analyzed in our previous work, chimerism occurs only in a small part of the hotspots. Whether the MDA generated chimeras have hotspot preferences, and how a preference forms, are not well understood. Characteristic exploration of hotspot selection is helpful for optimizing the reaction condition of MDA and might be beneficial for reducing chimeras.

In this study, we systematically screened the chimeric hotspots in the human genome and elaborately analyzed the chimeric hotspot selection in chromosome distribution, overlap length, overlap GC content and genomic distance between the two segments. Conjoint analyses of pairwise parameters were performed for the deep investigation of selection preferences. Through our analysis of millions of chimeric reads, regularly hotspot selective preferences were revealed in overlap length, GC content and distance. Selective preferences in overlap length and GC content exhibited to be pertinent with the sequence denaturation temperature. Our study revealed the profile of hotspot selective preferences of chimeric sequences through millions of chimeras and billions of chimeric hotspots in the human genome. The results might be helpful for optimizing MDA reaction conditions and reducing chimeras.

## 2. Materials and Methods

### 2.1. Data Source, Sample Information and Experimental Procedure

The human genome Hg19 was downloaded from the University of California, Santa Cruz (UCSC) genome browser [17]. Genome sequencing data were downloaded from the National Center for Biotechnology Information (NCBI) Sequence Read Archive (SRA) database; the SRA number was SRX252522. The type of sequencing data was paired-end 101 (PE–101).

The sequencing data were generated by Kaper et al. [9] in whole genome haplotyping analysis. In their study, genomic DNA sample NA18507 obtained from Coriell Cell Repositories was used for sequencing. The genomic DNA was diluted to 0.4 haploid copies per 1 µL water. A total of 1 µL was distributed into wells of 96-well microtiter plates. After denaturation, aliquots received hexamer primers and φ29 DNA polymerase. The reactions were incubated for 90 min at 30 °C and heat inactivated for 3 min at 65 °C. The final MDA products were used to generate sequencing libraries based on Nextera v2 technology according to the manufacturer’s protocol (Illumina, San Diego, CA, USA). Indexed whole-genome libraries were sequenced on a HiSeq2000 using 101-cycle paired-end, dual-indexing sequencing.

### 2.2. Recognition of Chimeras

The chimeras whose chimerism happened in the gap of the pair-end reads were excluded because the overlaps are indefinite. In order to get normalized information, avoiding the disruption of insertion and deletion, only the major type chimeras, 1-level chimeras with two inverted segments on complementary strands, were used for chimera recognition and further analysis (Figure 1).

The recognizing pipeline of 1-level chimera with two inverted segments on complementary strands followed the workflow described in our previous work [13]. After removal of the PE-101 raw reads with N, the clean reads were mapped to the Hg19 human genome reference by using SOAP2 software in single-end (SE) mode. The SE-101 mapped reads were abandoned, and the unmapped reads were kept for the following analysis. We cut 30 nt seeds from the kept reads and constructed a series of SE-30 reads. These reads were also mapped to the Hg19 human genome reference by using SOAP2 software in SE mode. In the alignment output data, the unmapped reads were abandoned while the others were kept for further analysis. According to the reads ID, we founded the single-end 101 (SE–101) reads from the SE-101 unmapped reads with the following characteristic: their 30-nt seeds could accurately map to the genome, while they could not on the same location. These reads would be collected as the candidate reads for the following detailed analysis of the chimeras with two segments on complementary strands. For collected candidate reads, the mapped 30-nt seeds were extended one nucleotide by one nucleotide on their related whole reads until they arrived at a mismatch site. Two segments of chimeras and the start-end coordinates of former segments on the reference genome were obtained. Afterwards, the following segments of chimeras were locally aligned in the 5 knt local region both upstream and downstream from the end coordinate of the former segments in order to find their exact location in the genome. After getting start-end coordinates of the following segments of chimeras, we reversely extended the following segments one nucleotide by one nucleotide from their start coordinates on the reference genome, and compared the nucleotide type with the end of the former subsections in order to obtain overlap sequences. The chimeras with two direct sequences that are located on the same strand were excluded because sequences with insertion or deletion might disrupt this kind of chimera. To obtain normalized information, only 1-level chimeras with two inverted segments on complementary strands were used for further analysis.

### 2.3. Chimeric Hotspots Screening in Hg19

Whole genome chimeric hotspot screening was performed to screen sequence pairs that have the potential to generate chimeras in MDA procedure. The screening pipeline was designed according to the characteristic of 1-level chimera with two inverted segments on complementary strands. Because the genomic distance of the two segments is mainly within 5 knt, we screened the sequence pairs whose sequences are reverse complementary with each other within 5 knt. Sequences from 3 to 25 nt were screened in the whole genome of Hg19. Because the amounts of reverse complementary single-nucleotide and dinucleotide pairs are extremely huge, we screened 1–2 nt hotspots only in chromosome 10. The amounts of these hotspots in the whole genome were calculated by multiplying the length folds between chromosome 10 and the whole genome.

When screening the 3-nt hotspots, we cut the first three nucleotides of a chromosome as a seed, and searched 3-nt segments whose sequences are reverse complementary with the seed in the next 5 knt. Then we slid the seed for one nucleotide, and searched for reverse complementary sequences in the next 5 knt of the new seed. The seed was slid over one nucleotide by one nucleotide until the length of the left sequence was smaller than 5 knt. The screening results of all chromosomes were recorded for further analysis. Other chimeric hotspots with different overlap lengths were screened following the same pipeline.

### 2.4. Analysis of Chimeras and Chimeric Hotspots

Chromosome distribution of chimeras and chimeric hotspots were depicted based on the chimeras and hotspot locations. Chromosome lengths were modified by removing the undetermined bases. The numbers of chimeras and chimeric hotspots were sorted by different overlap length and counted separately.

The overall average GC content and the average GC content of different overlap lengths were calculated separately by averaging the GC content of each conformable chimera or chimeric hotspot. In order to discover the diversity between the chimeras and chimeric hotspots, ΔGC between average GC content of chimera overlaps and average GC content of chimeric hotspot overlaps was calculated in different overlap lengths separately.

The distance between the two parts of the chimera was defined as the distance between end coordinates of the former segments and start coordinates of the following segments, and has been described in our previous work [13]. We constructed the following distance calculation, shown in Equation (1), to calculate the distance between two segments of chimeric hotspots based on the equation designed for chimeras.

*D = P*_seed,__end_*− P*_reverse__complementary__sequence,__start_ (1)

In the equation, *D* indicates the distance between the two sequences, *P*_seed,_ _end_ indicates the end coordinates of seeds, and *P*_reverse_ _complementary_ _sequence,_ _start_ indicates the start coordinates of the reverse complementary sequences.

The amounts of chimeras and chimeric hotspots in the distance from 0 to 5 knt were counted, taking 20 nt as one distance step.

### 2.5. T_d_ Estimation

The denaturation temperatures *T*_d_ of oligonucleotides were estimated using the classical Wallace-Itakura rule (2) [18,19]. *T*_d_ is the temperature at which one half of the duplexes are dissociated [18].

*T*_d_ (°C) *=* 4(*C* + *G*) + 2(*A* + *T*), (2)

## 3. Results

### 3.1. Chimeras and the Formation of Chimeras

Chimeras are chimeric rearranged DNA in amplified DNA of MDA [12]. The tendency to generate chimeric DNA rearrangements in amplified DNA is a major difficulty of MDA. A chimera normally consists of two rearranged segments. Chimeras consisting of three or more segments have also been discovered [13]. Chimeras consisted of two rearranged segments are called 1-level chimeras (Figure 1). Chimeras consisted of three or more rearranged segments are called 2-level or high level chimeras. Two or more segments of a chimera are discovered to be inconsecutively but adjacently located in genome [12,13]. According to our previous work, 1-level chimeras are the major chimeras, making up about 96% of all chimeras [13]. Distances between the joined segments of a chimera are mainly <5 knt [13]. At least two types of chimeric rearrangements were discovered in 1-level chimeras [12]. In the first type of rearrangement, two segments from complementary strands are rearranged, generating chimeras containing two inverted sequences. In the second type of rearrangement, two segments from the same DNA strands are rearranged, generating chimeras containing two direct sequences. More combined types occur in high level chimeras. Chimeras containing two inverted sequences are revealed to be the majority, about 2.4–5.7 times more than chimeras containing two direct segments [12,13]. Moreover, for chimeras containing two direct segments, the two segments are mapped to a same strand and the discrimination of chimeras might be disrupted by sequence insertion and deletion. Almost all chimeras have an overlapped sequence in the tail of the former segment and the head of the following segment (Figure 1). Sequence information of overlaps is important for recognizing chimerism. In next generation sequencing results, most PE reads contain an undetermined gap between the two ends. For chimeras whose combining site is located in the undetermined gaps, overlap sequences cannot be determined [13]. Hence, to get normalized information, only 1-level chimeras with two converted segments and a determined overlap were used in our work.

The accurate mechanism of the rearrangements is unclear. It is estimated to occur when displaced 3′-termini are freed to prime on nearby displaced 5′-strands [12]. MDA proceeds through a strand displacement mechanism with φ29 DNA polymerase, extending 3′-termini while concurrently displacing any downstream copies starting from their 5′-ends [20]. In the amplification, branched DNA molecules form and numerous single stranded 5′-ends and 3′-ends are generated. The mispriming of 3′-termini generate chimeras [12,13].

### 3.2. Screening Results of Chimeras and Chimeric Hotspots

In total, 36.7 million chimeras were classified as 1-level chimeras with two inverted segments on complementary strands. The average GC content of the chimera overlaps is 39.02%. Overlaps distribute from 1 to over 25 nt. A tiny part of the chimeras do not have overlap (0.4%) between the two segments. Meanwhile, by screening the whole genome of Hg19, about 195 billion chimeric hotspots from 3 to 25 nt in overlap length were obtained. The average GC content of the hotspot overlaps is 33.90%.

### 3.3. Chimeras and Hotspots Distribution in Chromosomes

**Figure 2:**
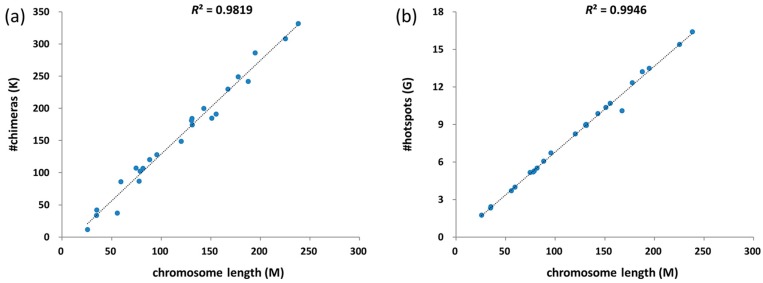
Scattergraph of number of chimeras or hotspots and chromosome lengths. (**a**) Scattergraph of number of chimeras and chromosome lengths. The number of chimeras in chromosome X and Y are doubled in the illustration because the other chromosomes are diploid; (**b**) Scattergraph of number of chimeric hotspots and chromosome lengths.

The total number of chimeras in different chromosomes is linearly associated with chromosome length (Figure 2a). The number of chimeras in chromosomes X and Y are doubled in Figure 2a, because the other chromosomes are diploid. After screening the whole genome, the amounts of chimeric hotspots containing 3–25 nt long overlaps exhibit a linear correlation with chromosome lengths (Figure 2b). The amounts of chimera discovered in different genes are also linearly associated with gene length (Figure S1). At least one chimera was observed in 80.9% genes (22,058 of 27,279 genes).

### 3.4. Chimeras and Hotspots Distribution in Overlap Length

**Figure 3:**
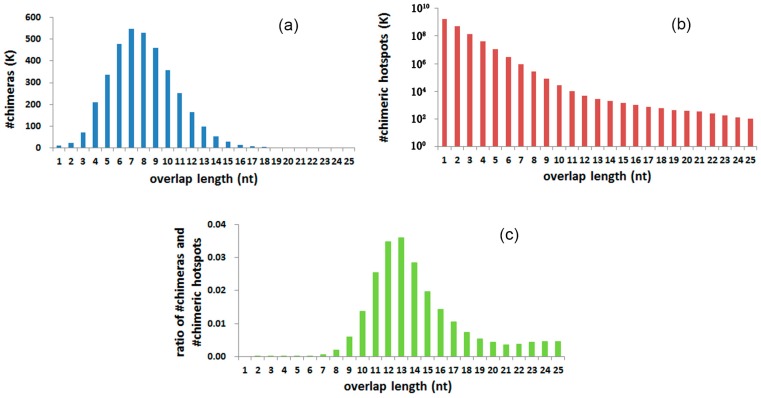
Chimera and chimeric hotspot distribution of overlap length. (**a**) Absolute number of chimeras with different overlap lengths; (**b**) Absolute number of chimeric hotspots with different overlap lengths. Hotspots for 1–2 nt were only screened in chromosome 10, and the numbers representing the whole genome were calculated by multiplying the size difference between chromosome 10 and the whole genome; (**c**) The ratio between the number of chimeras and hotspots with different overlap lengths.

The number of chimeras was counted in different overlap lengths, from 1 to 25 nt (Table 1, Figure 3a). The distribution of overlap lengths reveals a peak at 7 nt. Over 95% of overlaps are 3–13 nt long. The amount of chimeric hotspots in the genome declines as the overlap length increases (Figure 3b). For hotspots containing short overlaps, the amount declines to approximately 25% while the length increases one nucleotide. The declination is observed to be steady and gradual as the overlap length increases (Figure S2). Ratios between chimera numbers and chimeric hotspot numbers were calculated in overlap length, from 1 to 25 nt separately (Figure 3c). A peak in 12–13 nt is revealed after the calculation. The ratio tends to be stable when overlaps are longer than 19 nt.

### 3.5. GC Content and T_d_ of Overlaps

**Figure 4:**
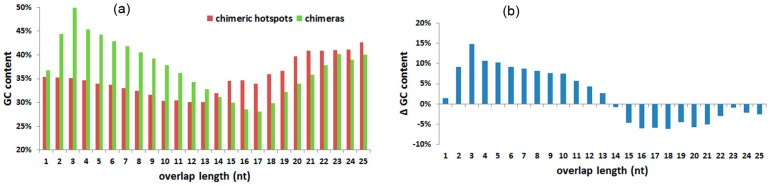
Average overlap GC content in overlap length. (**a**) Average overlap GC content in chimeras and chimeric hotspots in overlap length; (**b**) ΔGC between chimeras and chimeric hotspots in different overlap lengths.

The average GC content of chimera overlaps are shown in Table 1. GC content increases notably from 1 to 3 nt, the decreases gradually from 3 to 17 nt, and increases again after overlaps longer than 17 nt. The highest GC content of chimera overlaps is 49.88%, observed at 3 nt, while the lowest is 28.05% at 17 nt. The average GC content of overlaps contained in hotspots rank from 30.04% to 42.69%, revealing a valley at 12 nt (Figure 4a). ΔGC shows that smaller overlaps (≤13 nt) in chimeras have higher GC content than in hotspots (Figure 4b). For overlaps longer than 13 nt, the GC content of overlaps in chimeras are lower than in hotspots. The denaturation temperatures of overlap sequences were estimated and are shown in Table 1; they appear to be increasing with overlap extension.

### 3.6. Distance Distribution

**Figure 5:**
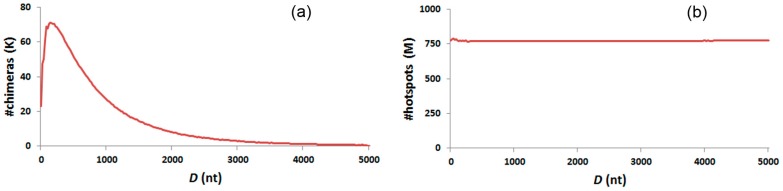
Chimera and chimeric hotspot distribution in distance of two segments. This scale takes 20 nt as one distance step. (**a**) Chimera distribution in distance of two segments; (**b**) Chimeric hotspot distribution in distance of two segments.

Distances between two segments of chimeras were calculated. Figure 5a shows chimera abundance with different distances, and exhibits a peak in chimera abundance from 80 to 280 nt. This distribution feature is also demonstrated by the chimeras of distinct overlap length (Figure S3a). Distances between two segments of chimeric hotspots seem to be equally distributed from 0–5 knt (Figure 5b). No locational specificity between the two segments is noticed. This distribution feature is also demonstrated by chimeric hotspots with distinct overlap length (Figure S3b).

## 4. Discussion

Due to the high processivity and proofreading activity of φ29 polymerase [10], MDA is considered to be a conventional approach and has been widely applied in comprehensive amplification from low input DNA. However, this isothermal amplification inevitably generates uneven amplifications across a genome and results in amplification errors [21,22,23]. Although technological innovations have improved the fidelity of MDA [2,24,25,26], some amplification biases and errors are still impacting its sensitivity and reliability. Systematic GC content bias in genome coverage were observed in MDA products, a 4.4× decrease in mean coverage between GC content of 0.35 and 0.48 in a MDA single-neuron sample set [14]. Chimera formation is another problem that still affect MDA method to some degree.

MDA chimeras are rearranged DNA artifacts in amplified DNA. Before the advent of next-generation sequencing, chimeras were rarely discovered. The large amount of data generated by next-generation sequencing provides a chance to discover plenty of chimeras. Chimeras in MDA were first discovered and analyzed in 2007, from sequencing data using the 454 sequencing platform [12]. Due to the relatively low sequencing throughput, only 475 chimeras were discovered in that study. Later, over 40 million chimeric reads were discovered from 130 Gb human MDA sequencing data [13]. Next-generation sequencing platforms generate reads in the final data, from 101 bp (Illumina platform, single-end) to over 400 bp (454 sequencing platform). If a combining site of the two rearranged sequences locates within a sequencing read or in the gap of a pair-end read, this read is considered to be a chimeric read. The bioinformatic recognition process of the combining sites is described in the materials and methods section. To obtain normalized information on overlap length, the GC content and the distance between the two segments, only 1-level chimeras with two inverted segments on complementary strands are used in this study. In order to study the selective preference of chimeras, chimeric hotspots screening was performed on the whole genome. The screening pipeline was designed according to the characteristic of 1-level chimera with two inverted segments on complementary strands.

A short overlap sequence of two segments is the core characteristic of chimeras. It suggests that most chimeras are produced by the rearrangement of two sequences which have a reverse complementary overlap. A pair of reverse complementary segments in sequence is defined as a chimeric hotspot, and has been systematically screened in human genome. By comparing the overlaps of all hotspots in the genome and the chimeras in sequencing data, significantly selective preferences come out, revealing some important information on MDA reaction and chimera generation. Selective preferences in overlap length and GC content manifest in direct relation to the denaturation temperature of the sequence. This indicates that the changes of denaturation factors influence the formation of chimeras, and points out an optimization direction to lessen chimera generation.

The number of hotspots is relevant to chromosome size, showing an equal distribution in the whole genome (Figure 2b). The equal distribution of chimeras indicates that no significant bias occurs during chromosome selection (Figure 2a). Not only hotspots but also chimeras are distributed among whole genome. The number of chimeras is relevant to the length of genes, revealing that the formation of chimeras is based on the DNA sequence and is not directly related to the functionality of genes (Figure S1).

The number of hotspots in the genome decreases as the overlaps elongate (Figure 3b). Among short overlaps (≤9 nt), about one fourth of the hotspots were screened in overlaps with one sequence 1 nt longer (Figure S2). The regular decrease reveals a general compliance with the random distribution. DNA sequence is composed of four kinds of nucleotides. Therefore, a sequence with 1 nt longer has a quarter of probability in finding the reverse complementary sequence of the shorter sequence. The mechanisms to explain why the ratio is a little more than 25% may be relative to the Chargaff second parity rule [27]. The ratios between adjacent long overlaps are abnormal, probably due to the presence of functional sequences and repeat sequences.

In considering hotspot selective preference in overlap length, the absolute quantities of chimeras are not suitable. The absolute quantities of chimeras with different overlap lengths are mainly codetermined by two factors; selective preference of the MDA reaction system and the amount diversity of the hotspots in genome. By calculating the ratio between the number of chimeras and the number of hotspots in different overlap lengths, the influence of quantity diversity of hotspots in a genome is excluded, and the selective preference is therefore revealed. The peak in Figure 3C indicates that the hotspots with overlaps of 12–13 nt are most likely to be selected as templates in the formation of a chimera generation. The maximum absolute number of chimeras is the number of chimeras containing 7-nt overlaps. This maximum number is mainly due to the huge number of hotspots of 7 nt, which is about 176 times greater than that of 12 nt. By conjoint analysis of overlap GC contents, the preference mechanisms of overlap lengths are revealed. Estimated *T*_d_ of overlaps of relatively abundant hotspots (12–13 nt) are approximately equal to MDA reaction temperature 30 °C (Table 1). This consistency indicates whether the reaction temperature and reaction conditions are appropriate for sequence denaturation and annealing, which determines the generation of chimeras. At least two steps are required to form a chimera. In the first step, a duplicated 3′ end denatures from the former sequence. In the second step, this free 3′ end anneals to a new sequence and misprimes the new sequence. Although ion concentration is also an influencing factor, the denaturation and annealing processes are mainly determined by temperature, length and GC content of a sequence [28]. At an assured temperature in MDA reactions, the sequences in the hotspots with high denaturation temperatures are not easy to denature from the former sequences, while the sequences in the hotspots with low denaturation temperatures are hard to anneal to new sequences. The estimated *T*_d_ of the most relatively abundant chimeras (12–13 nt) are a little higher than 30 °C. This is mainly due to the overestimating of the equation in long oligonucleotides [29]. In fact, the estimations of the denaturation temperature and the annealing temperature using different empirical formulas result in significant difference [30].

The selection bias in GC content also demonstrates whether the reaction conditions are appropriate for sequence denaturation and annealing, which again strongly influences the generation of chimeras. For overlaps shorter than 14 nt, chimeras are easy to be produced in the high GC content hotspots (Figure 4b). Short 3′-termini are easy to denature from the former sequences, but are hard to anneal to new sequences at the reaction temperature of MDA, due to their low annealing temperature. Hotspots with higher GC content have a higher annealing temperature based on the established sequence length. Hence, chimeras tend to be formed in the short hotspots with high GC content, because the 3′-termini are relatively easy to anneal to the new sequence at 30 °C, the reaction temperature of MDA. In contrast, for overlaps equal to or larger than 14 nt, low GC content hotspots are preferred in chimera generation. 3′-termini longer than 13 nt are hard to denature from the former sequences at 30 °C, the reaction temperature of MDA. 3′-termini with low GC content are relatively easy to denature from the former sequence and form free 3′ ends.

The number of hotspots decrease as the overlap length increases. For example, the number of hotspots whose overlaps are 13 nt is 27.3 million in the genome, and the number of hotspots with 14 nt overlaps is 19.3 million. If the rearrangement rate is certain, fewer chimeras will be generated in hotspots with 14-nt overlaps than in those with 13-nt overlaps. Hence, if the reaction condition is modified to be appropriate for longer sequence denaturation and annealing, fewer chimeras will be generated due to fewer hotspots containing longer overlaps in the genome. Therefore, modest modifications of reaction conditions may be beneficial to reduce chimeras. One of the modest modifications is to slightly raise the reaction temperature on the premise of keeping enzymes at the same activity level. If φ29 DNA polymerase has the same enzyme activity at 33 °C, this reaction temperature might be the optimal denaturation and annealing temperature for 13–14 nt 3′-termini to denature from the former sequence and anneal to a new sequence. The absolute number of hotspots with 13–14 nt overlaps is much smaller than the absolute number of hotspots with 12–13 nt overlaps. Therefore, the total number of chimeras ought to be smaller than what is obtained in this work.

Based on the probable generation process illustrated in our previous work [13], 1-level chimeras with two segments on complementary strands were generated by the jumping of 3′-termini from one sequence to another similar sequence. The similarity of the two sequences in nucleotide orders as well as the easiness of sequence denaturation and annealing at the reaction conditions influences the jumping probabilities. However, whether the distance of the two sequences influences the jumping is not clear. After analysis, the distances between the two segments of chimeras exhibit a peak at 80–280 nt (Figure 5a). The peak is not influenced by the amount of hotspots in sequence distance, since the distances between the two segments of hotspots are equally distributed in the genome (Figure 5b). Our results support that 3′-termini tend to jump from one sequence to another for 80–280 nt away in the genome (Figure 5a). This is probably because, within a distance of 80–280 nt, two sequences are most likely to locate adjacently or alternatively be captured by the same φ29 polymerase. For distance longer than 280 nt, the number of chimeras decreases smoothly. However, the number of chimeras fall sharply for distance shorter than 80 nt. Based on the linearity random coil structure of a single strand of DNA, the diversity of jumping probability between different spacing sequence lengths is reasonable. Longer, linear DNA may also coil, but the probability of one part of the sequence appearing nearby another similar part of the sequence falls with the elongation of spacing length. The sharp decline in shorter distances is probably due to the acutely increased difficulty in suitable coil, and may also result from the increased physical space occupied by the φ29 polymerase.

The characterization of MDA chimeras is helpful in distinguishing MDA chimeras. These characteristics include the existence of overlap, overlap length distribution of chimeras, and the distribution of the distance between the two segments of chimeras. Most chimeric sequences caused by disease do not have an overlap between two segments and the distance between two segments is restricted to within 5 knt. Therefore, the MDA chimeras can be distinguished and removed from the chimeric sequences caused by disease.

## 5. Conclusions

We systematically screen chimeric hotspots in the human genome and analyze the chimeric hotspot selection in over 36 million chimeras. Selective preferences in overlap length and GC content reveal a relation to the sequence denaturation temperature, indicating that the changing of denaturation-related factors will influence the formation of chimeras, which points out a direction to optimize the reduction of chimeras. The distance preference between two segments of chimeras at 80–280 nt provides a blurry image of the DNA sequence and φ29 DNA polymerase in physical space. The analysis presented here is beneficial for reducing chimera production, and the characterization of MDA chimeras is helpful in distinguishing MDA chimeras from chimeric sequences caused by disease.

## Figures and Tables

**Table 1:** Average GC content and estimation denaturation temperature of overlaps in chimeras.

Overlap Length (nt)	Number of Chimeras	Average GC Content	*T*_d_ (°C)
1	11,992	36.77%	2.74
2	22,292	44.40%	5.78
3	71,262	49.88%	8.99
4	208,729	45.31%	11.62
5	336,932	44.26%	14.43
6	476,409	42.84%	17.14
7	547,136	41.82%	19.86
8	528,555	40.53%	22.49
9	459,454	39.19%	25.05
10	356,470	37.82%	27.56
11	253,356	36.17%	29.96
12	163,502	34.35%	32.24
13	97,992	32.71%	34.51
14	54,711	31.08%	36.70
15	28,218	29.93%	38.98
16	14,630	28.59%	41.15
17	7429	28.05%	43.54
18	4189	29.79%	46.73
19	2310	32.13%	50.21
20	1661	33.99%	53.60
21	1200	35.80%	57.04
22	932	37.78%	60.62
23	740	40.11%	64.45
24	587	38.96%	66.70
25	480	40.10%	70.05

## Supplementary Materials

Supplementary materials can be found at www.mdpi.com/1422-0067/18/3/492/s1.
