# Supplementary material for: Hotspot Selective Preference of the Chimeric Sequences Formed in Multiple Displacement Amplification
Source: Int J Mol Sci. 2017 Feb 24;18(3):492. doi: 10.3390/ijms18030492 (PMC5372508; doi:10.3390/ijms18030492)
Supplement: Supplementary file 1 [file ijms-18-00492-s001.pdf]

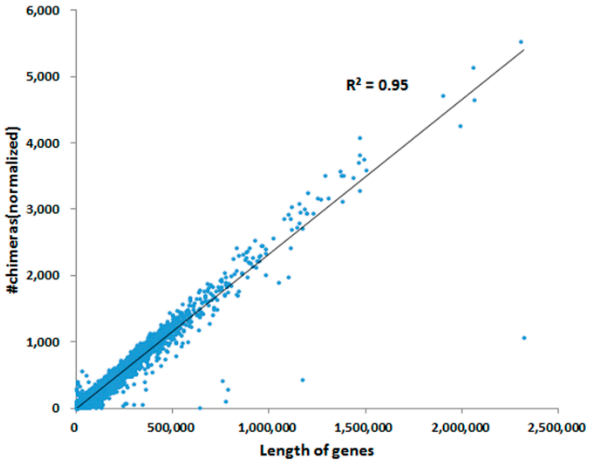


**Figure S1.** Scattergraph of normalized chimera numbers and gene lengths. The chimera numbers were normalized based on the sequencing depth of genes, to 30-fold of all genes. The genes which gotten at least five chimeras were drawn on this scattergraph.

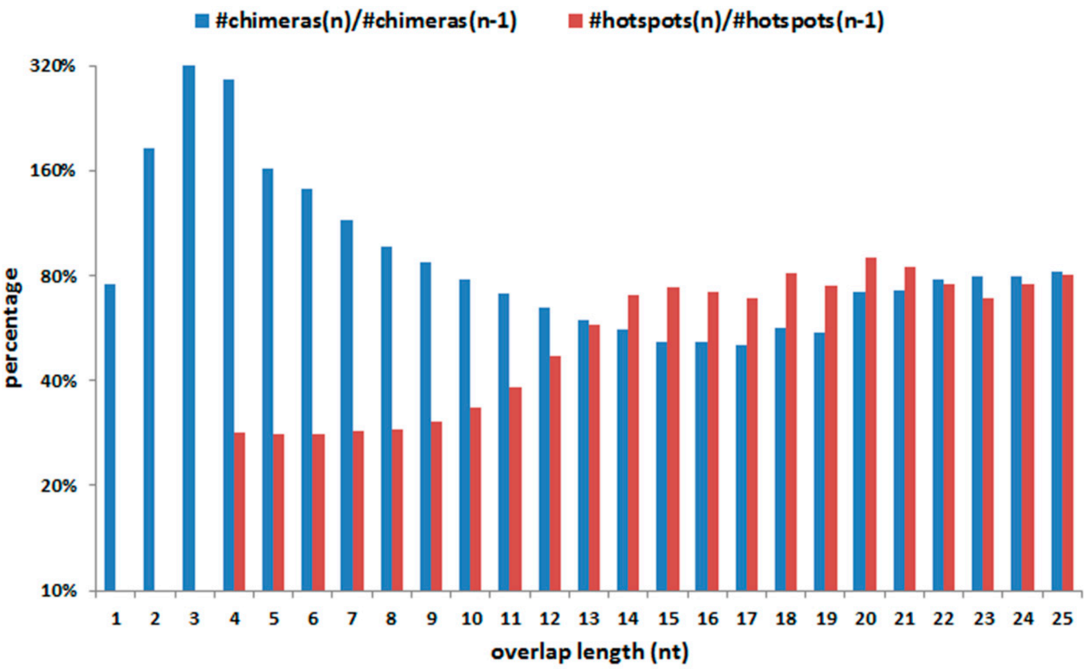


**Figure S2.** Ratio of the chimera or chimeric hotspots amounts between a given length overlap and 1 nt shorter overlap.

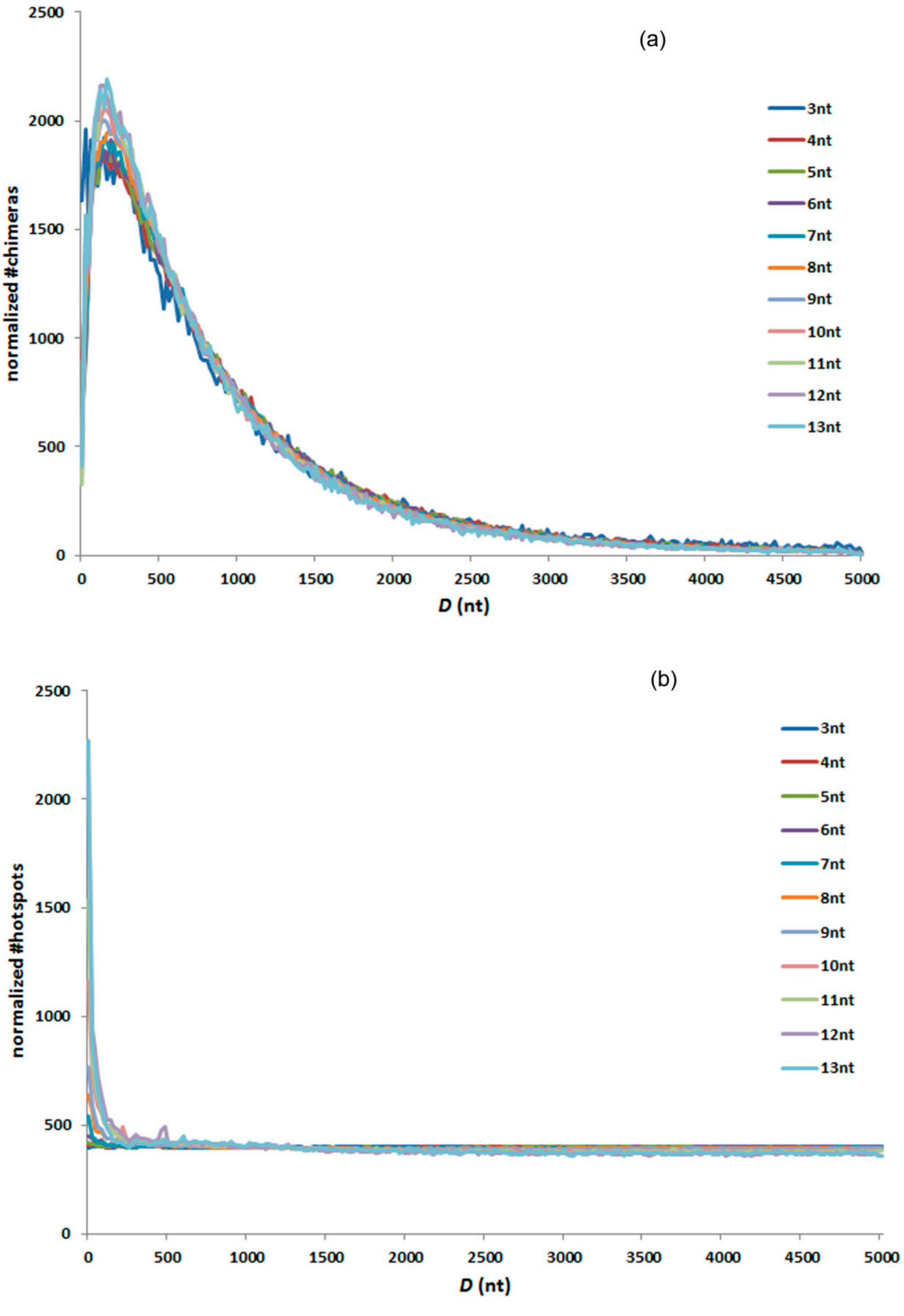


**Figure S3.** Distance distribution in different overlap lengths. (**a**) Distance distribution of chimeras in different overlap lengths. (**b**) Distance distribution of chimeric hotspots in different overlap lengths.
